# Supplementary material for: Evidence-Based View of Safety and Effectiveness of Prokineticin Receptors Antagonists during Pregnancy
Source: Biomedicines. 2021 Mar 17;9(3):309. doi: 10.3390/biomedicines9030309 (PMC8002561; doi:10.3390/biomedicines9030309)
Supplement: Supplementary file 1 [file biomedicines-09-00309-s001.pdf]

Evidence-Based View of Safety and Effectiveness of Prokineticin Receptors Antagonists during Pregnancy

Deborah Reynaud, Frederic Sergent, Roland Abi Nahed, Wael Traboulsi, Constance Collet, Christel Marquette, Pascale Hoffmann, Gianfranco Balboni, Qun-Yong Zhou, Padma Murthi, Mohamed Benharouga

Figure S1: Effects of prokineticin antagonists' PC7+PKRA on gestation outcomes

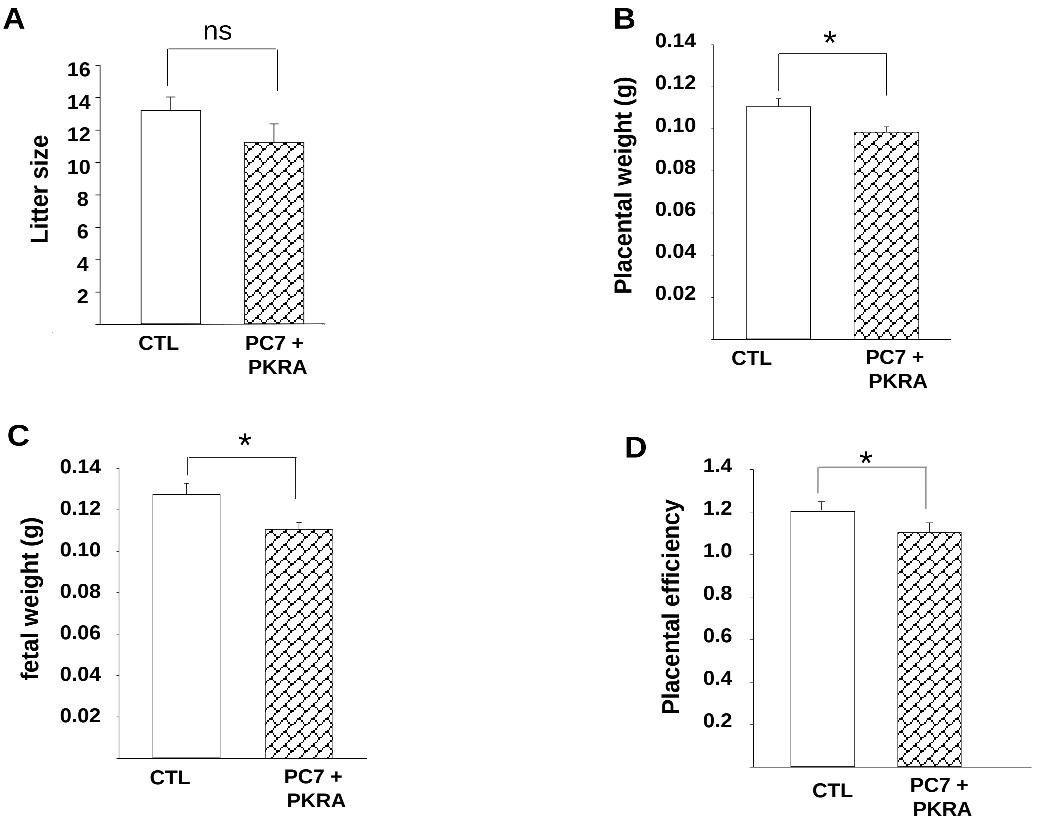

Figure S1. Effects of prokineticin antagonists' PC7+PKRA on gestation outcomes Panel B reports a graph that compares litter size of gravid mice treated or not with PC7+PKRA antagonists . Panel E & F report graphs that compare placenta and fetal weights of pups born from mother treated or not by PC7+PKRA, respectively. Panel D reports graph that compare placental efficiency of mice treated or not by PC7 or PKRA. Data are presented as mean ± SEM. * $p < 0.05$, bars with different letters are significantly different from each other.

Figure S2: Effects of combination concomitant treatment by prokineticin antagonists' on the placenta proliferation, vascularization and structure.

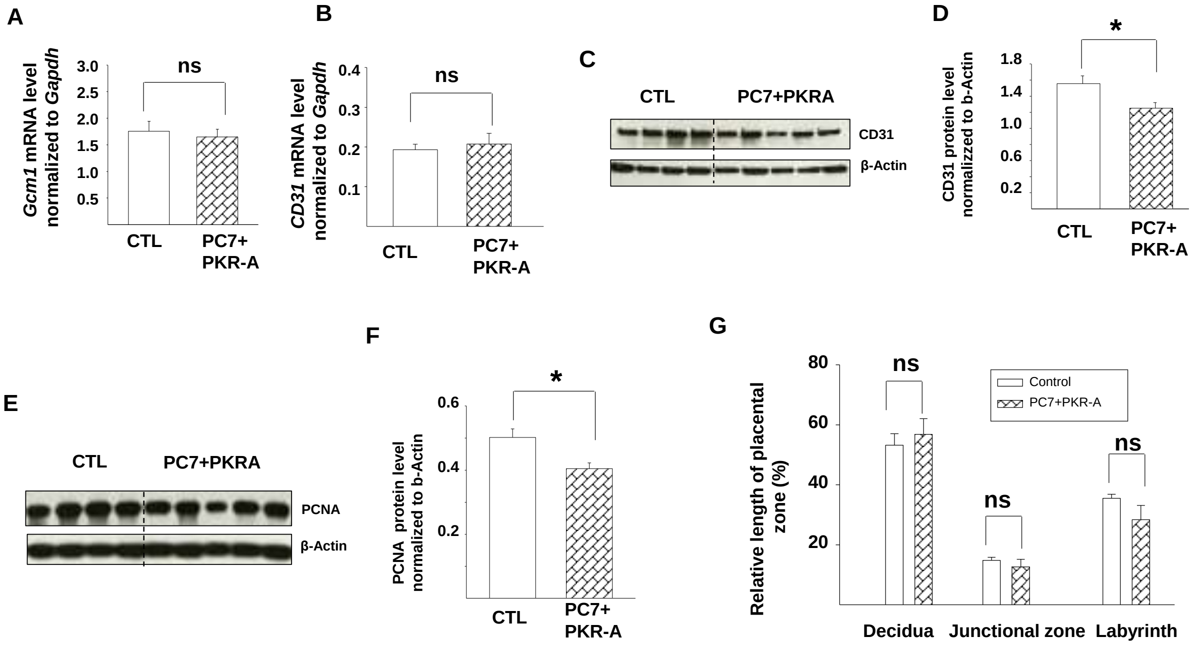

Figure S2. Effects of combined treatment by prokineticin antagonists' on the placenta proliferation and vascularization. **Panel A & B** depict comparisons of *Gcm1 and Cd31 mRNA* mRNA levels, respectively in placentas of CTL; PC7+PKRA. GAPDH was used to standardize for mRNA expression Data are represented as mean± SEM. ns = not significant, *P < 0.05. **Panel C & D** show Western blot analysis and quantification of CD31 protein levels in the placenta collected from CTL, PC7+PKRA treated mice. Standardization of immunoreactivity was performed using antibodies against β-actin.. **Panel E** show Western blot analysis and quantification of PCNA protein levels in the placenta collected from CTL and PC7+PKRA treated mice, **Panel F**. Standardization of immunoreactivity was performed using antibodies against β-actin. Data are represented as mean + SEM. * $p < 0.05$, ns = not significant. **Panel G** depicts analysis of the placental zones of CTL and PC7+PKRA placentas. For each group, three placental sections/animal were analyzed. The graph shows proportions of the surface layer of 3 zones of the placenta (labyrinth, junctional zone, and decidua). Surfaces of the 3 layers were measured on parasagittal sections for each placenta. Mean values were used to calculate the mean surface proportion of the layers. Data are represented as mean ± SEM. * $p < 0.05$, ns = not significant.

Figure S3: Effect of combination treatment by prokineticin antagonists' on trophoblasts invasion and differentiation.

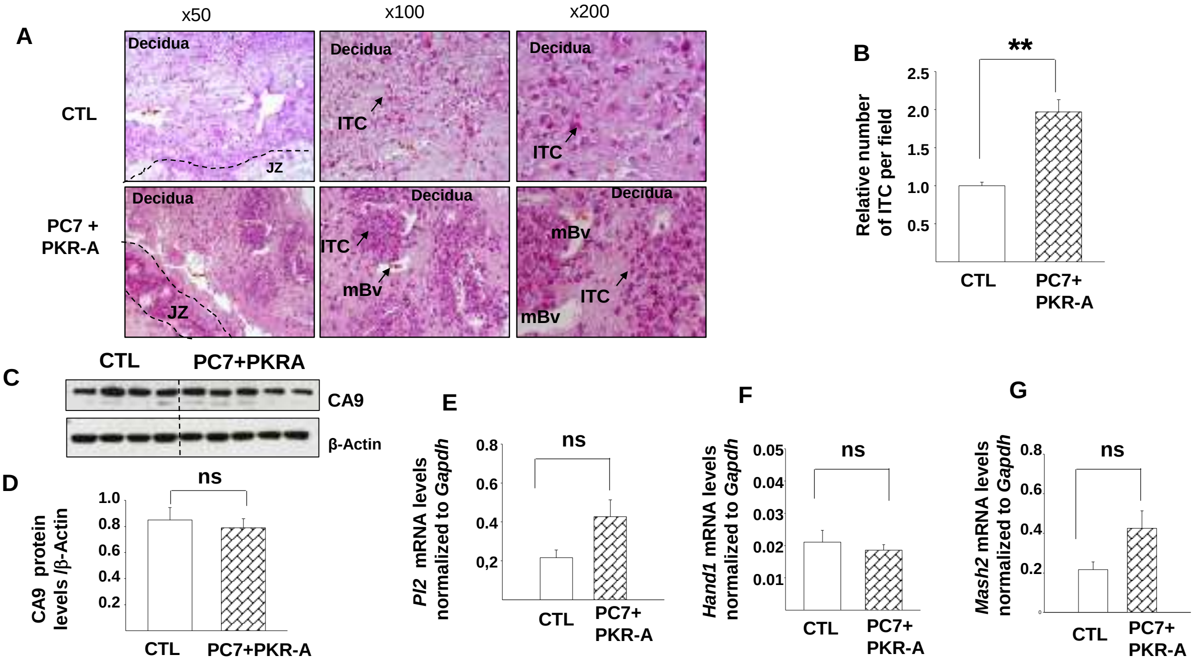

Figure S3. Effect of combined treatment by prokineticin antagonists' on trophoblasts invasion and differentiation. Panel A shows representative microphotographs of placental sections stained with Peroxide Acid Shiff at different magnifications. Placentas were collected from CTL, and PC7+PKRA treated mice at 12.5 dpc. **JZ**: Junctional Zone; **ITC**: Invasive Trophoblast Cell; **mBv**: Maternal Blood Vessel. **Panel B** depicts a graph that compares the number glycogenic cells in the maternal decidua. **Panel C & Panel D** show western blot analysis and quantification of CA9 protein levels in the placenta collected from CTL and PC7+PKRA treated mice. Standardization of immunoreactivity was performed using antibodies against β-actin. **Panels E, F & G** depict comparisons of *Placental lactogen*, *2 Hand1* and *Mash2* nd mRNA levels, respectively in placentas of CTL, PC7+PKRA. GAPDH was used to standardize for mRNA expression Data are represented as mean± SEM. ns = not significant, * $p < 0.05$.
